# Burdened and fatigued: the hidden costs of supporting undocumented students in postsecondary contexts

**DOI:** 10.3389/fpubh.2025.1644643

**Published:** 2025-09-23

**Authors:** Blanca Elizabeth Vega

**Affiliations:** Department of Educational Leadership, Montclair State University, Montclair, NJ, United States

**Keywords:** higher education and student affairs (HESA) professionals, immigration battle fatigue, undocumented students, administrative burden theory, brief research report

## Abstract

Sociopolitical and institutional barriers significantly influence the mental health and overall well-being of undocumented and Deferred Action for Childhood Arrivals (DACA) students in US higher education. Concurrently, higher education and student affairs (HESA) professionals who serve these students face their own psychological and professional challenges as they navigate restrictive policies and bureaucratic uncertainty. This brief research report extends the Immigration Battle Fatigue (IBF) framework by integrating principles from administrative burden theory to examine the interconnected experiences of undocumented students and the professionals who support them. Drawing on qualitative data from a Spencer Foundation–funded study of HESA professionals’ work with undocumented students, the analysis focuses on the psychological costs of immigration-related administrative burdens across four ecological levels: policy, institutional, interpersonal, and individual. Findings indicate that these psychological costs contribute to cumulative trauma, disengagement, and professional fatigue. The report concludes with implications for institutional practice, professional development, and policy reform to mitigate harm and promote systemic well-being.

## Introduction

In 2022, 32% or 5.8 million postsecondary students were either born outside the US or had at least one immigrant parent (1). Further, undocumented students comprise 409,000 individuals in higher education, a decrease from 2019, when 427,000 were reportedly enrolled (2). With more than a third of postsecondary students impacted by their immigration status, researchers, policymakers, and educational administrators need to prioritize understanding and addressing how immigration shapes the postsecondary experience.

Immigration-related factors significantly influence students’ postsecondary trajectories by affecting their overall health and well-being. Sixty percent of postsecondary students have been identified with one mental health concern (3), and over 75% reported psychological distress (33). While, in general, immigrant students arrive in the US with better health conditions than the US-born population, the stressors they experience (e.g., political climate) in the US change rapidly (4). As of this writing in 2025, the wave of raids and anti-immigration legislation imposed by the 47th administration has caused a significant number of immigrant people to report fear and distress related to national policymaking. Other stressors include restrictive immigration policies, causing mental health distress, such as anxiety and depression (5, 6). Researchers have also demonstrated that institutional agents in schools and postsecondary institutions can exacerbate the well-being of immigrant students, causing them to experience microaggressions (7), restrict access to pertinent information regarding college access and success, and even impart misinformation (34, 35, 48).

Institutional agents in postsecondary education include Higher Education and Student Affairs (HESA) professionals, who are administrators, educators, and leaders who support student learning, development, and success outside the classroom through roles in areas such as advising, student life, multicultural affairs, residence life, and career services (8, 9). They help shape campus climate, policies, and programs to foster equitable and inclusive educational environments (10, 11). Despite growing concerns about undocumented students’ well-being, research indicates that Higher Education and Student Affairs (HESA) professionals are perceived to lack awareness or care for the unique challenges these students face (12), and in some cases, may unintentionally inflict harm through microaggressions (7). At the same time, scholars have documented that HESA professionals themselves experience compassion fatigue when working with undocumented students, highlighting a need to address their mental health and emotional well-being (13) while others document the very real restrictions school agents experience when supporting undocumented students that constrain their ability to provide adequate support (36). This paper explores the intersection of these two strands of scholarship, the experiences of undocumented students and the psychological toll on HESA professionals, as a critical site of analysis.

In this brief research report, I examine the experiences of Higher Education and Student Affairs (HESA) professionals who work with undocumented students, alongside the policies shaping their practice. Drawing on qualitative data from a Spencer Foundation–funded study, I analyze the psychological costs of immigration-related administrative burdens across four interconnected levels: policy, institutional, interpersonal, and individual. This ecological perspective draws on Bronfenbrenner’s (47) assertion that human development is shaped by “the progressive, mutual accommodation between an active, growing human being and the changing properties of the immediate settings in which the person lives, as this process is affected by relations between these settings, and by the larger contexts in which the settings are embedded” (p. 21). Applying this framework highlights that these levels do not operate in isolation but interact to jointly shape the experiences and well-being of both students and professionals. By mapping these burdens across student and administrator pathways, the analysis underscores how systemic exclusion is embedded within and expressed across these layers. I argue that the psychological costs borne by undocumented students are closely tied to the administrative conflicts HESA professionals face within a complex and often hostile federal policy environment. Findings reveal that these costs contribute to cumulative trauma, disengagement, and secondary fatigue. I conclude by outlining implications for institutional practice, professional development, and policy reform aimed at mitigating harm and fostering systemic well-being.

## Overview of relevant research

Postsecondary students whose educational trajectories are shaped and constrained by the federal government’s use of immigration status as a criterion for determining eligibility for resources face a distinct set of challenges as they navigate the educational pipeline. This section briefly examines the experiences and well-being of DACA recipients and undocumented students at the postsecondary level. While the literature underscores the central role of Higher Education and Student Affairs (HESA) professionals in supporting the well-being of undocumented students, existing scholarship primarily reflects the perspectives of the students themselves, offering limited insight into the experiences and practices of the HESA professionals who work with them as part of this critical relationship.

### Overview of undocumented students in higher education

While immigrant students experience distinct legal liminalities, for this paper, I will focus on students who are undocumented (i.e., individuals who do not have the documentation to provide them with protection from deportation). Undocumented immigrants are individuals born outside of the United States who reside within its borders without legal authorization (14). Within the context of higher education, undocumented students are those who similarly lack legal immigration status yet pursue postsecondary education in the U. S. These students often have lived in the United States for the majority of their lives, are fluent in English, and may have been unaware of their undocumented status until adolescence or early adulthood. Notably, both undocumented immigrants and undocumented students lack a clear and accessible pathway to citizenship (15, 37).

Diverse legal liminalities are important to highlight in this section. Recipients of the Deferred Action for Childhood Arrivals (DACA) are also considered undocumented, but with certain protections. DACA is an executive order established in 2012 under President Obama’s administration. It has served 834,000 individuals, and despite its success, it faced attacks during the 45th administration, with efforts to rescind it beginning in 2017. In January 2025, the U. S. District Court for the Southern District of Texas ruled that a DACA decision was unlawful and issued a partial stay, allowing DACA recipients who applied in 2021 to renew their applications but not accepting new applicants. Nine states (Texas, Alabama, Arkansas, Kansas, Louisiana, Mississippi, Nebraska, South Carolina, and West Virginia) were plaintiffs in this ruling.

Despite these legal and structural limitations, all children, regardless of immigration status, are entitled to attend public elementary and secondary schools, as guaranteed by the Plyler v. Doe (16) decision. However, this legal protection does not extend to postsecondary education. At the federal level, undocumented students are barred from accessing most forms of postsecondary benefits, including federal financial aid. Nevertheless, some states have enacted policies that mitigate these restrictions by applying state residency criteria to extend in-state tuition and, in some cases, state-based financial aid to undocumented students.

### Undocumented student health in higher education

Given the lack of protection undocumented students face in higher education, it is not surprising that they face a distinct set of health-related challenges. Legal precarity, limited access to health services, and chronic psychosocial stress are factors that shape undocumented student health (17). Undocumented students navigate a complex and often hostile policy environment while attempting to meet academic and personal expectations, frequently navigating enforced invisibility within institutional life (17). A growing body of research underscores that both mental and physical health concerns are prominent in this population, alongside significant barriers to care and wellness access (18, 19).

**Mental Health Strain and Psychosocial Stress**. Undocumented students report elevated levels of anxiety, depression, and chronic stress relative to their documented peers, often linked to fear of deportation, institutional discrimination, and financial precarity (17, 20). Cha et al. (20) found that these stressors are frequently internalized and normalized, leading many students to interpret their psychological distress as an inevitable outcome of undocumented status, thereby diminishing help-seeking behaviors. Perceptions that mental health interventions are ineffective in addressing systemic causes, such as immigration-related exclusions, further discourage service use. The intersectional stigma associated with both undocumented status and mental illness adds to these barriers, creating significant psychosocial obstacles to care. In many cases, students avoid seeking help out of concern that they will be perceived as weak or risk disclosing their immigration status, even in confidential therapeutic settings.

**Federal and State Barriers to Health Care Access**. Lack of health insurance remains one of the most substantial barriers to care. Most undocumented students are ineligible for Medicaid, the Children’s Health Insurance Program (CHIP), and coverage under the Affordable Care Act (ACA) (21), leaving them reliant on emergency care or informal support networks. DACA recipients became eligible to enroll in ACA programs under the Biden administration in 2024. However, the expanded access was challenged by 19 states, preventing DACA students from enrolling in health care assistance in those states. Even when campus or community health services are technically available, fears of institutional surveillance, lack of trust, and concerns about confidentiality contribute to underutilization (20).

**Immigration Policy and Campus Climate**. Shifting immigration policy landscapes, such as threats to the Deferred Action for Childhood Arrivals (DACA) program, generate constant instability for undocumented students and exacerbate mental health risks (22). Uncertainty over legal protections has been linked to heightened anxiety and emotional distress. However, research highlights that inclusive, supportive institutional environments or “undocu-serving” campuses can serve as a critical protective factor, promoting psychological well-being, academic persistence, and a greater sense of belonging (23).

### The role of higher education and student affairs professionals in the well-being and mental health of DACA and undocumented students

While some universities offer mental health training for HESA professionals (24), this training is not mandatory, nor is it consistently included in HESA graduate programs. As a result, many professionals enter the field without a solid foundation in mental health or a nuanced understanding of how immigration status intersects with student well-being. Although mental health services may be available on campus, they are not always equipped to serve students with diverse and liminal immigration statuses effectively, especially when services are hard to access. In response, some institutions have developed specialized support programs, such as culturally responsive counseling, peer support groups, and wellness initiatives for international and undocumented students (25). Community partnerships with local health providers and social services have also become vital resources for students facing financial hardship or complex legal issues.

Despite these efforts, the role of HESA professionals is often limited to service provision and student support, neglecting the emotional labor they perform and the institutional gaps they must navigate. Research indicates that HESA professionals who work closely with undocumented students may suffer from compassion fatigue or secondary trauma due to repeated exposure to students’ immigration-related stressors, worsened by a lack of training and institutional support (13). Furthermore, the literature underscores the possibility and responsibility of HESA professionals to act as institutional change agents. By leveraging their strategic roles, these professionals can help shape inclusive campus climates that resist and mitigate the effects of exclusionary immigration policies (26, 27). For example, after the 45th presidential administration rescinded DACA in 2017, university leaders have reassured undocumented students of their safety on campus by affirming that Immigration and Customs Enforcement (ICE) officials are not permitted to conduct arrests on university grounds. During that time, many university leaders have publicly denounced the 45^th^ administration’s actions on DACA.

Nevertheless, the limited attention to immigration status among higher education leadership and other HESA professionals remains evident. Public statements by university leaders have often been inconsistent and, in some cases, have conveyed harmful or ambiguous messages regarding immigration enforcement, border control, and the roles of undocumented parents (27). As stated earlier, research on undocumented students’ perceptions of HESA professionals further underscores these challenges, with several studies documenting negative interactions. Vega et al. (38) demonstrate that such interactions can produce significant psychological and behavioral consequences, contributing to immigration battle fatigue—often exacerbated by the very professionals charged with supporting undocumented students—which compounds their already precarious circumstances. To interrogate these tensions more fully, this brief research report draws on a conceptual framework incorporating immigration battle fatigue, immigrant illegality, and administrative burden theory. Together, these frameworks reveal how HESA professionals can either perpetuate or disrupt institutional inequities and, in doing so, profoundly influence the well-being of undocumented students in postsecondary education.

## Conceptual framework

This study draws on three intersecting frameworks—Immigration Battle Fatigue, Immigrant Illegality, and Administrative Burden Theory—to examine how undocumented students in higher education and the Higher Education and Student Affairs (HESA) professionals who support them experience and navigate immigration-related stressors. Together, these frameworks illuminate how exclusionary policies, institutional practices, and social perceptions converge to produce cumulative psychological, emotional, and behavioral strain for both groups.

Situated within an ecological perspective spanning four levels—policy, institutional, interpersonal, and individual—this approach emphasizes the relational nature of these burdens. Immigration Battle Fatigue (38), adapted from Smith’s (28, 29) concept of Racial Battle Fatigue, highlights the ongoing stress, reduced motivation, and withdrawal behaviors that arise when undocumented students face persistent policy confusion, stigma, and institutional neglect. Immigrant Illegality (39) describes undocumented status as a socially and legally constructed condition that limits access to resources, even in environments with seemingly inclusive policies, due to ongoing stigma and administrative ambiguity (30, 31). Administrative Burden Theory (32) enhances this analysis by identifying the learning, compliance, and psychological burdens embedded in policy and institutional design—burdens that undocumented students must manage and that HESA professionals often face when advocating without sufficient institutional authority or guidance (40).

By integrating these frameworks within an ecological model, this study captures the complexity of how policy, institutional structures, and human relationships interact to shape everyday experiences. This approach moves beyond single-actor or single-level analyses, instead recognizing that students’ and professionals’ pathways are intertwined in ways that both reflect and resist systemic exclusion. In doing so, it positions the empirical analysis to not only document harm but also identify leverage points for institutional and policy interventions that promote equity and well-being.

## Methods

### Research questions and methods

Building on the integrated ecological framework outlined above, this study employs a qualitative, multi-site case study design to examine how Higher Education and Student Affairs (HESA) administrators navigate the complex interplay of federal, state, and institutional politics surrounding undocumented students. The analysis is guided by the following research question: How do HESA administrators navigate conflicting federal, state, and university policies related to undocumented students?

### Sample and site selection

While federal policy has remained largely static in restricting undocumented students’ access to postsecondary benefits, state-level responses vary considerably. These policy contexts can be grouped into three broad categories:

1. Inclusive: Tuition Equity States: States offering in-state tuition and/or state-based financial aid to undocumented students.
2. No State Policy: States with no explicit legislation regarding undocumented students’ access to higher education benefits, but where some of their public institutions may have implemented inclusive practices toward undocumented students.
3. Prohibitive/Restrictive States: States with policies banning or limiting undocumented students’ enrollment (prohibitive), in-state tuition eligibility (restrictive), or access to state financial aid (restrictive).

Thirty participants were interviewed across 15 states and Washington, DC, reflecting a wide range of institutional types and policy climates. The sample included higher education and student affairs (HESA) administrators from international student services, diversity and multicultural affairs, academic advising, legal services, and offices dedicated to undocumented student support, as well as higher education leaders in admissions, enrollment management, and student success initiatives. Most participants worked directly with undocumented students, while others engaged through advising, programming, advocacy, or policy interpretation.

Demographically, the sample was predominantly female (80%) and racially and ethnically diverse, with the majority identifying as Latina/o, Chicana/o, or Hispanic (60%). White/Caucasian participants comprised 20% of the sample, with additional representation from Asian (7%), Black/African American (3%), Native American/Latino (3%), and Jewish diaspora (3%) participants.

Participants were situated in states with varying policy climates: 70% in inclusive contexts (e.g., New Jersey, California, Illinois), 17% in prohibitive contexts (e.g., Georgia, South Carolina), 3% in restrictive contexts (North Carolina), and 10% in states with no statewide policy (Pennsylvania and Michigan, where access is limited to certain public universities). Institution types spanned large public HSIs (50%), medium public HSIs (13%), small private liberal arts colleges (13%), community colleges (10%), large public non-HSIs (10%), and private selective universities, including law and Ivy institutions (10%). Collectively, these participants offered insights into how state and institutional contexts shaped practices, policies, and advocacy for undocumented students in higher education. Table 1 shares participants’ demographics and institutional contexts.

**Table 1:** Participant demographics and institutional contexts (*N* = 30).

Category	Subcategory	n	%
Gender identity	Female / She (incl. She/ella, she/ella/)	24	80%
	Male / He (incl. he/el, him/his)	6	20%
Race/Ethnicity	Latina/o/Chicana/o/Hispanic	18	60%
	White / Caucasian	6	20%
	Black / African American	1	3%
	Asian	2	7%
	Native American/Latino (mixed heritage)	1	3%
	Jewish diaspora	1	3%
State policy climate	Inclusive (e.g., NJ, IL, CT, NY, OR, MA, CA, NV, DC, VA, TX*)	21	70%
	Restrictive (e.g., North Carolina)	1	3%
	Prohibitive (e.g., GA, SC)	5	17%
	No State Policy (e.g., PA, MI**)	3	10%
Institution type	Large Public HSIs (incl. EHSI/AANAPISI)	15	50%
	Medium Public Institutions (HSI/EHSI)	4	13%
	Small Private Liberal Arts (HWI/HSI)	4	13%
	Community Colleges (public/HSI/CC)	3	10%
	Large Public (non-HSI specified, e.g., land grant)	3	10%
	Private (large, selective, law/Ivy)	3	10%

*Texas is categorized as “Inclusive until 2025” when the policy was changed. **Pennsylvania and Michigan are classified as “No State Policy.” Access and tuition equity are limited to certain public universities that have adopted their own inclusive practices.

### Data collection

Data were gathered through 30 in-depth, semi-structured interviews conducted via Zoom between 2021 and 2024. Interviews lasted between 60 and 90 min and were initially transcribed using automated transcription software. Transcripts were then verified against the audio recordings for accuracy and returned to participants for member checking.

In addition to interviews, I collected and analyzed state-level policy documents, legislative summaries, institutional websites, annual reports, and relevant media coverage to develop detailed state policy profiles. These profiles informed the interview protocols and provided critical context for interpreting participants’ accounts (41). Data collection concluded prior to the policy shifts initiated by the 47th presidential administration in January 2025.

### Recruitment of participants

Drawing on my professional networks as a former HESA administrator and current faculty member in a higher education program, I recruited participants using purposeful and snowball sampling (43). I sought “positioned informants” (42) or individuals whose professional roles afforded them insight into how policy and politics shaped institutional practice. Interview questions explored participants’ knowledge of federal and state guidelines, strategies for implementation, and how personal beliefs, organizational priorities, and political contexts informed their decision-making.

### Data analysis

Data analysis proceeded in multiple stages, beginning with the development of contextual state profiles to capture variation in policy environments. State profiles were constructed from legislative documents, institutional policies, and public statements and categorized into the three policy contexts described above. These profiles provided an analytic scaffold for comparing institutional and professional practices across policy environments. Interview transcripts were analyzed inductively using a multi-level coding process (44). In the first coding cycle, descriptive and *in vivo* codes were applied to capture participants’ own language and surface-level patterns in their narratives. This stage identified broad topical areas such as *policy interpretation*, *student trust*, *emotional exhaustion*, and *institutional advocacy*.

In the second coding cycle, focused coding was employed to synthesize and refine these preliminary codes into analytically robust categories aligned with the study’s conceptual framework. Codes were organized across the four ecological levels (i.e., policy, institutional, interpersonal, and individual) and connected to themes such as administrative burden, policy conflict, and professional well-being.

Throughout the process, analytic memos were written after each interview to document emerging insights, link patterns to the ecological framework, and note possible contradictions. Using constant comparative and cross-case analysis (45), I examined how political, institutional, and local factors interacted within and across policy contexts. All coding and analysis were conducted using Dedoose software (Table 2).

**Table 2:** Sample codebook.

Code	Description	Example Quote	Ecological level	Policy context
Policy context	References to state policy environment and its perceived inclusivity or restrictiveness	“We’re in a restrictive state, it’s unclear what we are even allowed to do.”	Policy	Prohibitive/Restrictive
Motivation for work	Participant’s personal or professional reasons for supporting undocumented students	“I was undocumented myself, and I saw the need in my community.”	Individual	All
Student information access	How undocumented students obtain or fail to obtain college-related information	“They usually hear about in-state aid from peers, not staff.”	Institutional	Inclusive – Tuition Equity
Office conflict	Tensions between departments or offices regarding undocumented student policies	“Financial aid says one thing, our office says another, and the student is caught.”	Institutional	No State Policy
Stressors	Emotional or psychological strain from supporting undocumented students	“It’s exhausting to support students when you feel helpless and alone.”	Individual	All
Trust building	Efforts to establish trust with undocumented students	“They only share their status when they know they can trust you.”	Interpersonal	All

### Researcher positionality

I have studied the experiences of immigrant students in higher education for over 25 years, beginning my work with undocumented students in 1998. My research has documented the critical role HESA administrators play in providing accurate information on college access and success—from tuition and financial aid guidance to navigating mental health resources. Despite this role, administrators often have little to no formal training on serving undocumented students, yet they bear the responsibility of implementing related policies and practices. Grounded in Dillard’s (46) call for research as a responsibility to the communities we engage with, this study centers the voices of HESA administrators to better understand how they navigate these complex policy landscapes.

## Findings overview

The findings are organized according to the four ecological levels—policy, institutional, interpersonal, and individual—and interpreted through the three state policy contexts identified in the state profiles: Inclusive—Tuition Equity States, No State Policy States, and Prohibitive/Restrictive States. While thematic patterns emerged across all contexts, their scope, severity, and mechanisms varied. In inclusive states, in-state tuition and state aid reduced some financial barriers but did not eliminate stigma, administrative ambiguity, or the emotional labor required to navigate higher education. In states with no policy, the absence of legislative guidance created institutional inconsistency and left decision-making to individual offices or leaders. In prohibitive/restrictive states, statutory exclusion from enrollment or tuition, combined with hostile political rhetoric, produced acute fear, discouraged disclosure, and intensified psychological costs for both undocumented students and the HESA professionals who support them.

### Policy level: conflicting mandates and layered exclusions

Across all contexts, participants described the mental toll of policy contradictions where one level of governance offered a pathway to access while another imposed barriers. This reflected the administrative burden’s learning and psychological costs: students and professionals struggled to understand and emotionally process complex and changing rules. For example, in *Inclusive–Tuition Equity States*, while state policies allowed for in-state tuition or aid, students remained ineligible for federal benefits such as financial aid, creating what one administrator called “half-open doors.” Professionals described the challenge of explaining these “yes, but…” scenarios without discouraging students. In *No State Policy States*, the lack of explicit policy created a vacuum in which institutional leaders either stepped in with *ad hoc* guidance or avoided the issue altogether. This left HESA professionals without a standard playbook, increasing uncertainty and internal policy conflicts. Finally, in *Prohibitive/Restrictive States,* statutory bans on in-state tuition or admissions are layered on top of federal exclusions, producing an Immigrant Battle Fatigue (IBF) effect. HESA professionals reported that undocumented students experienced chronic stress, fear of exposure, and avoidance of opportunities, leading professionals to operate under a “compliance over care” mandate.

Zara, a professional in a state that, as of 2018, became inclusive, shared her experiences working there amid the threats posed by the DACA rescission (2017) and the incoming 45^th^ administration in 2016. She highlighted the psychological toll:

“We were told that at any point… the government could just come through our doors and ask for files. We needed to be prepared for that… We are not going to allow this to happen to our students.”

Zara recalled that the preparation to support students did not include training and involved protecting students’ identities. She recalled fear associated with this time, even though she worked within a state that offered in-state tuition. She not only feared for her students but also feared for her colleagues.

### Institutional level: organizational ambiguity and advocacy constraints

Institutional policies and norms either mitigated or amplified state-level dynamics, embodying immigrant illegality when practices reinforced exclusionary logic. For participants in Inclusive States, their campuses often publicized support services, but inconsistent training led to students still encountering misinformation. Some offices acted as strong advocates, while others perpetuated confusion by relying on outdated or incomplete policy knowledge. For administrators in *No State Policy* states, without legislative guardrails, institutional responses depended on campus leaders who were aware of the well-being of undocumented students and their sense of safety on campus. This created supportive spaces or HESA professionals within campuses but left students vulnerable to variable interpretations and procedural roadblocks.

In *Prohibitive/Restrictive* states, participants shared that they had to defer to the most restrictive reading of policy, adding internal barriers beyond those required by law. For example, one participant shared that many institutions in prohibitive states not only prevented undocumented students from in-state tuition and financial aid, but some campuses also required all non-resident students to enroll for classes after the general population enrolled. Professionals described “working around” these systems quietly to avoid drawing political attention.

Participants in inclusive, tuition-equity states faced a similar challenge: working around these systems while also being responsible for creating systems and training. Sara, from a tuition-equity state, shared that she did not receive formal training from her campus and often sought guidance from community-based organizations. She was responsible for sharing this knowledge with her colleagues. She described the informal strategies they developed: “There wasn’t a training … we kind of just taught each other how to make sure [undocumented students] were safe and supported.” Tina, also in an inclusive, tuition-equity state, emphasized the importance of nuanced categorization in advocacy work: “I separate [students] by employment authorization, DACA recipients, or TPS asylum individuals, and students who could not apply to DACA … cannot change their immigration status.”

### Interpersonal level: trust as selective currency

Relationships between students and staff reflected the interpersonal transmission of administrative burden—where trust, once established, eased navigation, but the scarcity of safe relationships concentrated emotional labor on a few individuals.

Participants from Inclusive States shared that students were more willing to disclose status to designated staff or cultural centers, but this trust was still selective and had to be earned over time. This meant consistent outreach from participants to the general campus, to be “found.” Tina, in an inclusive state, explained her tailored approach:

“It’s a very different experience for undocumented students … seeing what they need and how to tailor those needs so they could be helped as soon as they walk in.” Campuses with free legal services (found in inclusive contexts) were able to operationalize trust-building into tangible protections. Participants often worked with lawyers or clinics on their campus, which created more centers of support and overt outreach.

Participants in *No State Policy* states found that disclosure was inconsistent, depending on students’ perception of a staff member’s discretion and how well it aligned with advocacy values. Professionals sometimes discovered a student’s status only after a crisis occurred. A similar pattern appeared in *Prohibitive/Restrictive* states, where disclosure was rare and typically happened in high-stakes situations (e.g., financial holds or absences from class). Staff seen as allies took on extra advising, emotional support, and problem-solving tasks. In restrictive states, such resources were limited or discreet, underscoring the uneven geography of institutional care.

### Individual level: coping, withdrawal, and professional exit

At the individual level, participants’ accounts reflected the embodied outcomes of IBF and immigrant illegality—showing decision fatigue, avoidance, and self-withdrawal among students, and burnout or role abandonment among professionals. In Inclusive States, professionals expressed concern about undocumented students’ ability to persist in their studies, describing constant vigilance, especially regarding disclosing their status in classrooms or applications. Many participants in this study also engaged in advocacy, particularly those who are immigrants or were undocumented, but worried about the long-term emotional toll. Nonetheless, all participants in inclusive states recognized that their colleagues in restrictive or prohibitive states faced tougher challenges and preferred to work within inclusive states. Despite these layered feelings, participants were concerned about leaving their roles, fearing that their work would leave with them and create a vacuum.

For participants in *No State Policy* states, uncertainty fostered frustration and fear. Professionals described a feeling of “treading water” without institutional support, and some questioned whether their roles could be sustained. In Prohibitive/Restrictive states, participants noted that ongoing exclusion and hostile environments created hopelessness among students and demoralization among advocates, who often considered leaving the institution or sector entirely. However, all professionals acknowledged the privilege of mobility—the ability to move to safer spaces—a privilege their students could not access, highlighting the disparities across different settings.

## Discussion

An ecological perspective on Immigration Battle Fatigue (IBF) provides a framework for understanding how exclusionary systems function through policy, institutional inertia, and interpersonal tension. It emphasizes the dual burden on students and Higher Education and Student Affairs (HESA) professionals, as well as the urgent need for coordinated campus responses. These burdens result from policy design and administrative choices.

These findings underscore the complex and multifaceted nature of psychological costs in higher education for both undocumented students and the HESA professionals who support them. At every level—policy, institutional, interpersonal, and individual—the experiences of HESA professionals who work with undocumented students reveal the cumulative effects of administrative burden and immigration-related fatigue.

At the policy level, conflicting mandates and exclusionary practices create ongoing fear and confusion, fueling psychological distress. Zara’s detailed accounts of the threats caused by federal policies during the Trump administration illustrate how these worries can deeply affect the work environment and the overall campus climate. Such policies lead to disengagement among students and burden staff who feel compelled to operate in a climate of fear and silence.

At the institutional level, the lack of consistent procedures and clear messaging worsens these challenges. HESA professionals like Sara described the absence of formal training and the patchwork methods they use to support students in crisis. These experiences align with findings that show how inconsistent institutional practices and unclear guidance lead to confusion, stress, and unfair outcomes for undocumented students (38). Additionally, the burden of advocacy and “invisible labor” performed by staff like Tina illustrates organizational practices that lead to burnout and staff turnover. Many of these HESA professionals do this work without recognition or staff support, which leaves them feeling helpless and unsure if their efforts are making a difference.

At the interpersonal level, trust and safety are key themes. Tina’s story about providing tailored support and free legal services on her campus emphasizes the importance of creating safe spaces for undocumented students. However, relying on individual staff members for trust-building also places a heavy emotional burden on those perceived as “safe,” which can lead to secondary trauma and compassion fatigue (13). Sara’s example of finding a workaround for stipends shows the innovative strategies HESA professionals use in the absence of institutional support, highlighting both their dedication and the unsustainable nature of this work.

The psychological impact of these cumulative barriers is clear at the individual level. Undocumented students reported feeling disengaged and withdrawing due to constant stress and uncertainty, aligning with previous research on decision fatigue and the internalization of illegality (38). HESA professionals also shared feelings of helplessness and burnout, questioning whether their work is sustainable in institutions that often lack structural support. As some participants noted, unlike staff who might leave for more supportive environments, undocumented students do not have the same mobility or privilege to leave, which deepens the inequalities involved.

## Implications and future directions

These findings demand urgent action on multiple levels. Policies that generate fear and confusion (such as in-state tuition exclusions and admissions bans) need to be challenged and reformed to guarantee equal access to education and lessen the psychological stress on students and staff. At the institutional level, universities must acknowledge the diversity within immigrant student populations and commit to comprehensive training, clear protocols, and structural support for undocumented students and their advocates. Trauma-informed practices, consistent messaging, and compensating staff for the unseen labor of advocacy are vital steps forward.

Further research should continue examining the dual burden of immigration-related fatigue on undocumented students and HESA professionals, especially amid changing political landscapes and policy shifts. Expanding the IBF framework to include intersectional identities (e.g., race, gender, and health status) would provide even deeper insights into the complex experiences of burden and fatigue. Ultimately, addressing these psychological costs requires fostering individual resilience, systemic reform in policy and campus structures, and professional practices to create truly equitable and safe educational environments for undocumented students.

## Data Availability

The datasets presented in this article are not readily available because participants were offered confidentiality. Requests to access the datasets should be directed to vegab@montclair.edu.

## References

[1] Higher Education Immigration Portal. (2022). State data. Available online at: https://www.higheredimmigrationportal.org (Accessed August 1, 2025).

[2] FWD.US (2023). The post DACA generation. Available online at: https://www.fwd.us/news/undocumented-high-school-graduates/ (Accessed August 1, 2025).

[3] Lipson SK Zhou S Abelson S Heinze J Jirsa M Morigney J. Trends in college student mental health and help-seeking by race/ethnicity: findings from the national healthy minds study, 2013–2021. J Affect Disord. (2022) 306:138–47. doi: 10.1016/j.jad.2022.03.038, PMID: 35307411 PMC8995361

[4] Dombou C Omonaiye O Fraser S Cénat JM Fournier K Yaya S. Barriers and facilitators associated with the use of mental health services among immigrant students in high-income countries: a systematic scoping review. PLoS One. (2023) 18:e0287162. doi: 10.1371/journal.pone.0287162, PMID: 37384726 PMC10310021

[5] Martinez O Wu E Sandfort T Dodge B Carballo-Dieguez A Pinto R. Evaluating the impact of immigration policies on health status among undocumented immigrants: a systematic review. J Immigr Minor Health. (2015) 17:947–70. doi: 10.1007/s10903-013-9968-4, PMID: 24375382 PMC4074451

[6] Ornelas IJ Yamanis TJ Ruiz RA. The health of undocumented Latinx immigrants: what we know and future directions. Annu Rev Public Health. (2020) 41:289–308. doi: 10.1146/annurev-publhealth-040119-094211, PMID: 32237989 PMC9246400

[7] Nienhusser HK Vega BE Saavedra Carquin M. Undocumented students’ experiences with microaggressions during their college choice process. Teach Coll Rec. (2016) 118:1–33. doi: 10.1177/016146811611800208

[8] Long D. The foundations of student affairs: a guide to the profession In: Hinchliffe LJ Wong MA, editors. Environments for student growth and development: Librarians and student affairs in collaboration. Chicago, IL: Association of College & Research Libraries (2012). 1–39.

[9] Wright-Mair R Saadeddine R Peters C Elmes A. Higher education and student affairs master's students' perceptions of their preparation for scholarly practice and implications for program improvement: a mixed methods case study. Coll Stud Aff J. (2022) 40:13–32. doi: 10.1353/csj.2022.0012

[10] Garcia CE Walker W Morgan D Shi Y. Aligning student affairs practice with espoused commitments to equity, diversity, and inclusion. J Coll Stud Dev. (2021) 62:137–53. doi: 10.1353/csd.2021.0013

[11] Pérez II D Ashlee KC Do VH Karikari SN Sim C. Re-conceptualizing student success in higher education: reflections from graduate student affairs educators using anti-deficit achievement framework. J Excell Coll Teach. (2017) 28, 5–28. doi: 10.1080/01419870.2022.2062251

[12] Luedke CL Corral D. Supportive or exclusive? Institutional agents and undocumented Latina/o college students in the Midwest. J Coll Stud Dev. (2021) 62:575–90. doi: 10.1353/csd.2021.0051

[13] Hoy ZRM Nguyen DHK. Compassion fatigue in student affairs practitioners working with undocumented college students. Coll Stud Aff J. (2020) 38:126–42. doi: 10.1353/csj.2020.0007

[14] Immigrants Rising. (2024). Defining undocumented [Handout]. Immigrants Rising. Available online at: https://immigrantsrising.org/resource/defining-undocumented/ (Accessed on June 8, 2025)

[15] Immigrants Rising. (2024). Overview of undocumented students [Handout]. Immigrants Rising. Available online at: https://immigrantsrising.org/resource/overview-of-undocumented-students/ (Accessed on June 8, 2025)

[16] Plyler. Doe, 457 U.S. 202 (1982). Available online at: https://www.oyez.org/cases/1981/80-1538 (Accessed August 1, 2025).

[17] Nienhusser HK Romandia O. Undocumented college students’ psychosocial well-being: a systematic review. Curr Opin Psychol. (2022) 47:101412. doi: 10.1016/j.copsyc.2022.101412, PMID: 35926397

[18] Garcini LM Daly R Chen N Mehl J Pham T Phan T. Undocumented immigrants and mental health: a systematic review of recent methodology and findings in the United States. J Migration Health. (2021) 4:100058. doi: 10.1016/j.jmh.2021.100058, PMID: 34405198 PMC8352099

[19] Suárez-Orozco C López Hernández G. “Waking up every day with the worry”: a mixed-methods study of anxiety in undocumented Latinx college students. Front Psychol. (2020) 11:568167. doi: 10.3389/fpsyt.2020.568167, PMID: 33281641 PMC7691235

[20] Cha BS Enriquez LE Ro A. Beyond access: psychosocial barriers to undocumented students’ use of mental health services. Soc Sci Med. (2019) 233:193–200. doi: 10.1016/j.socscimed.2019.06.011, PMID: 31212126

[21] Baumrucker EP Kolker AF. Noncitizen eligibility for Medicaid and CHIP (CRS In Focus No. IF11912). Hershey, Pennsylvania, USA: Congressional Research Service (2025).

[22] Dadras O Hazratzai MS. The silent trauma: US immigration policies and mental health. Lancet Regional Health–Americas. (2025) 44:100768. doi: 10.1016/j.lana.2025.100768 PMC11914743 40104491

[23] Salazar C Nader C Zuñiga JDV Viruel AR. Promoting culturally engaging campus environments for undocumented students: the role of institutional agents. Innovative Higher Educ. (2025) 50:1247–80. doi: 10.1007/s10755-025-09723-7

[24] Vega B. E. Talamo A. Coleman C. (2024). The importance of mental health training in faculty development. ACPA Developments. Available online at: https://developments.myacpa.org/private7J9pRkXzLq/29/the-importance-of-mental-health-training-in-faculty-development-vega-talamo-coleman/2345/ (Accessed August 1, 2025).

[25] Camilo D. Culturally responsive college application advising for undocumented immigrant students In: Rausch MA Gallo LL, editors. Strengthening school counselor advocacy and practice for important populations and difficult topics. Hershey, Pennsylvania, USA: Information Science Reference/IGI Global (2021). 62–84.

[26] Enriquez LE Vazquez Vera D Ramakrishnan SK. Driver's licenses for all? Racialized illegality and the implementation of progressive immigration policy in California. Law & Policy. (2019) 41:34–58. doi: 10.1111/lapo.12109

[27] Vega B. E. (2023). Undocumented students and related policies in region 2 states. NASPA Blog Available online at: https://www.naspa.org/blog/undocumented-students-and-related-policies-in-region-2-states (Accessed August 1, 2025).

[28] Smith WA. Black faculty coping with racial battle fatigue: the campus racial climate in a post-civil rights era In: Cleveland D, editor. A long way to go: Conversations about race by African American faculty and graduate students. New York, NY: Peter Lang (2004). 171–90.

[29] Smith WA Hung M Franklin JD. Racial battle fatigue and the miseducation of black men: racial microaggressions, societal problems, and environmental stress. J Negro Educ. (2011) 80:63–82.

[30] Del Real D. Seemingly inclusive liminal legality: the fragility and illegality production of Colombia’s legalization programmes for Venezuelan migrants. J Ethn Migr Stud. (2022) 48:3580–601. doi: 10.1080/1369183X.2021.1953501, PMID: 36061515 PMC9439259

[31] Menjívar C Agadjanian V Oh B. The contradictions of liminal legality: economic attainment and civic engagement of central American immigrants on temporary protected status. Soc Probl. (2022) 69:678–98. doi: 10.1093/socpro/spab017, PMID: 37649781 PMC10468158

[32] Herd P Moynihan DP. Administrative burden: Policymaking by other means. Russell Sage Foundation. (2019).

[33] American College Health Association. (2021). National College Health Assessment (NCHA): Reference group executive summary spring 2021. American College Health Association.

[34] Enriquez L. “Because we feel the pressure and we also feel the support”: Examining the educational success of undocumented immigrant Latina/o students. Harv Educ Rev. (2011) 81:476–500.

[35] Lauby F. “Because she knew that I did not have a social”: Ad hoc guidance strategies for Latino undocumented students. J Hisp High Educ. (2017) 16:24–42. doi: 10.1177/1538192715614954

[36] Díaz-Strong DX Moreno DG. Undocumented students’ uneven access to financial aid resources: How existing resources reinforce deservingness. Educ Policy Anal Arch. (2025) 33:Article 126. doi: 10.14507/epaa.33.8804

[37] Enyioha JC. College access for undocumented students and law. Educational Considerations. (2019) 45:7. doi: 10.4148/0146-9282.2168

[38] Vega BE Nienhusser HK Carquin-Hamichand MS. “When I would hurt”: Undocumented students’ responses to obstacles faced during the college choice process. Ed Forum. (2022) 86:237–252. doi: 10.1080/00131725.2021.2020944

[39] Enriquez LE. A “master status” or the “final straw”? Assessing the role of immigration status in Latino undocumented youths’ pathways out of school. J Ethnic Migr Stud. (2017) 43:1526–1543. doi: 10.1080/1369183X.2016.1245608

[40] Halling A Bækgaard M. Administrative burden in citizen–state interactions: A systematic literature review. J Public Adm Res Theory. (2023) 34:180–195. doi: 10.1093/jopart/muad019

[41] Yin RK. Case study research and applications: Design and methods (6th ed.). SAGE Publications. (2018).

[42] Kezar A. Reconstructing static images of leadership: An application of positionality theory. J Leadersh Stud. (2002) 8:94–109. doi: 10.1177/107179190200800308

[43] Patton MQ. Qualitative evaluation and research methods (2nd ed.). SAGE Publications. (1990).

[44] Saldaña J. The coding manual for qualitative researchers (3rd ed.). SAGE Publications. (2016).

[45] Miles MB Huberman AM. Qualitative data analysis: An expanded sourcebook (2nd ed.). SAGE Publications. (1994).

[46] Dillard CB. On spiritual strivings: Transforming an African American woman’s academic life. State University of New York Press. (2006).

[47] Bronfenbrenner U. The ecology of human development: Experiments by nature and design. Harvard University Press. (1979).

[48] Gonzales RG. Lives in limbo: Undocumented and coming of age in America. Univ of California Press. (2016).

